# *Diphyllobothrium nihonkaiense* Tapeworm Larvae in Salmon from North America

**DOI:** 10.3201/eid2302.161026

**Published:** 2017-02

**Authors:** Roman Kuchta, Mikuláš Oros, Jayde Ferguson, Tomáš Scholz

**Affiliations:** Biology Centre, Czech Academy of Sciences, České Budějovice, Czech Republic (R. Kuchta, T. Scholz);; Slovak Academy of Sciences, Košice, Slovak Republic (M. Oros);; State of Alaska Department of Fish and Game, Anchorage, Alaska, USA (J. Ferguson)

**Keywords:** foodborne disease, food safety, zoonoses, Alaska, Cestoda, plerocercoid, broad tapeworm, *Diphyllobothrium nihonkaiense*, salmon, North America, parasites

## Abstract

Diphyllobothriosis is reemerging because of global importation and increased popularity of eating raw fish. We detected *Diphyllobothrium nihonkaiense* plerocercoids in the musculature of wild pink salmon (*Oncorhynchus gorbuscha*) from Alaska, USA. Therefore, salmon from the American and Asian Pacific coasts and elsewhere pose potential dangers for persons who eat these fish raw.

The Japanese broad tapeworm, *Diphyllobothrium nihonkaiense* (Yamane, Kamo, Bylund et Wikgren, 1986) (Cestoda: Diphyllobothriidea) is the second most common causative agent of diphyllobothriosis in humans; ≈2,000 cases have been reported, mainly from northeastern Asia (*1*). However, recent studies that used molecular methods indicate that the number of human cases caused by this tapeworm may have been highly underestimated (*1*). In addition, increasing popularity of eating raw fish is probably responsible for the increased number of imported cases in regions where this infection is not endemic (*1*).

In 1986, the Japanese broad tapeworm was recognized as a human parasite separate from the most common broad fish tapeworm, *Diphyllobothrium latum*, in Japan (*2*). The validity of the Japanese broad tapeworm was later confirmed by molecular data, especially the *cox*1 gene sequences (*1*). Evidence indicates that virtually all previous cases of diphyllobothriosis in humans in Japan, South Korea, and the Pacific coast of Russia that were attributed to *D. latum* tapeworms were caused by *D. nihonkaiense* tapeworms (*1*,*3*). Moreover, *D. klebanovskii* (Muratov et Posokhov, 1988) from the Pacific coast of Russia was recently synonymized with the Japanese broad tapeworm (*1*,*4*).

Studies on the transmission of the Japanese broad tapeworm in Japan and eastern Russia (Primorsky Region) have identified 4 species of Pacific salmon as the principal sources of human infection: chum salmon (*Oncorhynchus keta*), masu salmon (*O. masou*), pink salmon (*O. gorbuscha*), and sockeye salmon (*O. nerka*). These anadromous fish become infected in brackish water along the coast of the North Pacific Ocean (*1*,*5*). Tapeworm larvae infective for humans (plerocercoids) have been described in only a few studies performed in eastern Russia and Japan, (e.g., as plerocercoids type F from the musculature of chum salmon in Kamchatka, Russia) (*2*,*6*,*7*).

For decades, the possible occurrence of the Japanese broad tapeworm on the Pacific coast of North America was ignored, but since 2008, human infection with adult tapeworms and natural infection of carnivores (wolves and bears) with adult tapeworms have been confirmed by use of molecular markers (*1*,*8*–*10*). We report finding Japanese broad tapeworm plerocercoids in North America. Our main intent is to alert parasitologists and medical doctors about the potential danger of human infection with this long tapeworm resulting from consumption of infected salmon imported (on ice) from the Pacific coast of North America and elsewhere.

In July 2013, we examined 64 wild Pacific salmon of 5 species:1 chinook salmon (*O. tshawytscha*), 1 coho salmon (*O. kisutch*), 23 pink salmon, 8 rainbow trout (*O. mykiss*), and 31 sockeye salmon in south-central Alaska, USA. The salmon were collected by angling (under permit no. SF2013–218) or obtained from local fishermen. The musculature was filleted to narrow slices, and internal organs were observed under a magnifying glass. Several morphotypes of diphyllobothriid plerocercoids were found, including a single larva in the musculature of pink salmon collected in Resurrection Creek (near Hope, Alaska). This plerocercoid, which was later identified as that of the *D. nihonkaiense* tapeworm, was found unencysted, deep in the musculature of the anterior part of the fish, near the spinal cord (Figure). It was highly motile, had a retracting scolex, and was 8–15 mm long, depending on the state of elongation or contraction (Figure; Video). After fixation with hot water, the plerocercoid was 10 mm long, had an elongate scolex 1.05 mm long and 0.60 mm wide, and possessed 2 narrow bothria opened on the apical end (Figure). The sequences of the *cox*1 and 28S rRNA genes (*lsr*DNA) were almost identical to those of the Japanese broad tapeworm available in the GenBank database (sequence similarities of 99% [GenBank accession no. KY000483] and 100% [KY000484], respectively), thus providing unequivocal support that this plerocercoid was a larva of the *D. nihonkaiense* tapeworm reported from North America.

**Figure:**
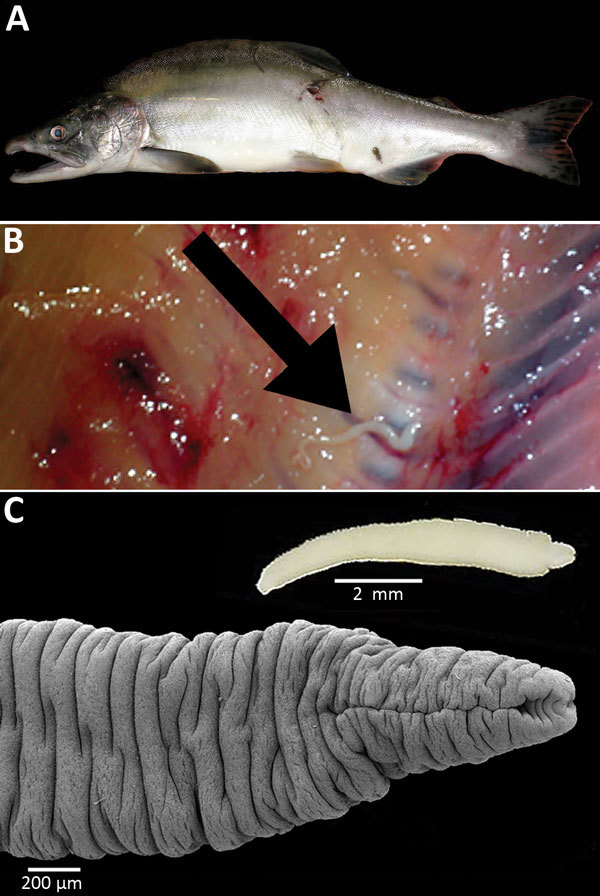
A) Pink salmon (*Oncorhynchus gorbuscha*) from Alaska, USA. B) Plerocercoid of Japanese broad tapeworm (*Diphyllobothrium nihonkaiense*) (arrow) deep in the muscles of the salmon. C) Live *D. nihonka* plerocercoid in saline (inset) and scanning electron micrograph after fixation with hot water; note the scolex with a long, slit-like bothrium opened anteriorly.

**Video:**
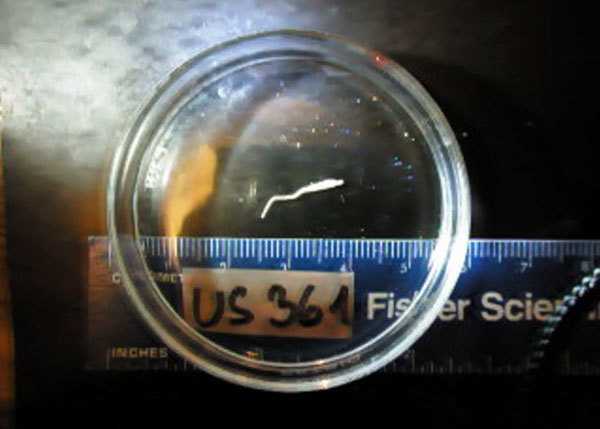
Live plerocercoid of the Japanese broad tapeworm (*Diphyllobothrium nihonkaiense*) obtained from the musculature of pink salmon (*Oncorhynchus gorbuscha*) from Alaska, USA.

This report provides additional evidence that salmon from the Pacific coast of North America may represent a source of human infection. Because Pacific salmon are frequently exported unfrozen, on ice, plerocercoids may survive transport and cause human infections in areas where they are not endemic, such as China, Europe, New Zealand, and middle and eastern United States (*1*). It is probable that most diphyllobothriosis cases originally attributed to *D. latum* may have been caused by *D. nihonkaiense* tapeworms. For more effective control of this human foodborne parasite, detection of the sources of human infection (i.e., host associations), and critical revision of the current knowledge of the distribution and transmission patterns of individual human-infecting tapeworms are needed.
